# Implementation of a patient‐reported experience measure in a Dutch disability care organization: A process evaluation of cocreated tailored strategies

**DOI:** 10.1111/hex.13628

**Published:** 2022-11-08

**Authors:** Marjolein van Rooijen, Anneke van Dijk‐de Vries, Stephanie Lenzen, Ruth Dalemans, Albine Moser, Anna J. H. M. Beurskens

**Affiliations:** ^1^ Department of Family Medicine, CAPHRI School for Public Health and Primary Care Maastricht University Maastricht The Netherlands; ^2^ Research Centre for Autonomy and Participation of Persons with a Chronic Illness Zuyd University of Applied Sciences Heerlen The Netherlands

**Keywords:** care user, cocreation, communication‐vulnerable, disability care, implementation

## Abstract

**Introduction:**

In 24/7 disability care facilities, patient‐reported experience measures (PREMs) are important to help healthcare professionals understand what matters to care users and to improve the quality of care. However, the successful implementation of a PREM is complex. In a Dutch disability care organization, stakeholders cocreated tailored implementation strategies aimed at improving the use and integration of a qualitative PREM. This study gives insights into the uptake and experiences with these cocreated implementation strategies and the perceived impact of the set of strategies.

**Methods:**

We performed a prospective process evaluation between February 2020 and February 2021. We collected data in three disability care facilities from 35 care users, 11 professionals, 3 facility managers and 4 organization representatives. Data collection included observations during kick‐offs and learning goal meetings and several attendance checklists. We collected 133 questionnaires (Time 0 and Time 1). We conducted 35 individual semistructured interviews and an online focus group interview. Quantitative data were analysed using descriptive statistics and qualitative data using directed content analysis.

**Results:**

The exposure to and adoption of strategies was between 76% and 100%. Participants were positive about tailoring the strategies to each facility. Implementation was hindered by challenges in care users' communication and COVID‐19. The perceived impact referred to an improved understanding of the goal and added value of the PREM and better preparation and execution of the PREM. The impact of the set of strategies was mainly experienced on the micro level.

**Conclusion:**

The uptake of the cocreated implementation strategies was acceptable. The participants valued the tailored approach, which enabled them to focus on facility‐specific learning goals. Stakeholder engagement and co‐created strategies may have strengthened the adoption of and experiences with the implementation.

**Patient or Public Contribution:**

In this article, we present the process evaluation of implementation strategies for the integrated use of a PREM in disability care. A development group consisting of communication vulnerable care users, trainers and professionals developed the implementation strategies. The disability care organization was responsible for the planning and organization of the implementation process. During the process evaluation the end users, trainers, professionals and managers tailored the implementation strategies to their own settings and needs. Researchers observed this implementation process and interviewed the stakeholders about their experiences and the perceived impact.

## 1 INTRODUCTION

Over the past 20 years, the importance of quality of care and its transparency has increased in the disability sector.^1^, ^2^ Next to measures of physical quality, such as malnutrition and medicine effectiveness, the emphasis is increasingly on care users' perceptions of their health status and quality of life, as measured by patient‐reported outcome measures, and on care users' experiences of the care, as measured by patient‐reported experience measures (PREMs).^3^, ^4^ Insights into care users' experiences can lead to more involvement of them in decisions about care preferences and to more effective relationships between them and healthcare professionals.^3^, ^5^, ^6^, ^7^, ^8^ PREMs are essential to understand what kind of support care users wish to make their life meaningful, especially in the disability sector, in which care users rely on 24‐h care.^9^ Most PREMs are structured questionnaires^10^; however, there are also more qualitative instruments that support a structured conversation between care users and professionals. The advantage of qualitative PREMs is their potential to uncover, in more depth, individual care users' experiences with the received care.^11^, ^12^, ^13^

In 2017, a new quality framework for the Dutch disability sector was introduced.^9^ This framework emphasises the integrated use of PREMs to improve the quality of care.^9^, ^11^ This implies that information about care users' experiences needs to include quality information on three levels: (1) the micro level (care user–professional level), to enhance the delivery of appropriate care and the development of individual care plans; (2) the meso level (organizational level), to monitor the quality of care and enhance team reflection and (3) the macro level, to facilitate organization‐wide improvements and external reporting about quality of care.

The successful uptake of a qualitative PREM in routine practice demands both collecting meaningful experiences of care users and integrated use of the outcomes at the micro, meso and macro levels. However, there are various challenges to successfully integrating a PREM into routine care. These challenges include proper preparation of implementation, an efficient work process and a doable way of entering outcomes in the electronic patient records and using the outcomes for quality reports.^11^, ^14^, ^15^, ^16^

Stichting Gehandicaptenzorg Limburg (SGL) is a Dutch disability care organization for people with acquired brain injuries. Many SGL care users experience communication vulnerability, which encompasses elements of speech, language, hearing disorders, gestures or semantics, resulting in experienced functional communication difficulties and difficulties in expressing themselves and in understanding professionals.^17^ SGL uses a qualitative PREM called ‘Dit vind ik ervan!’ (‘This is how I feel about it!’; see Supporting Information: Appendix I). This PREM facilitates a structured dialogue that encompasses 10 themes.^18^

**Figure 1:**
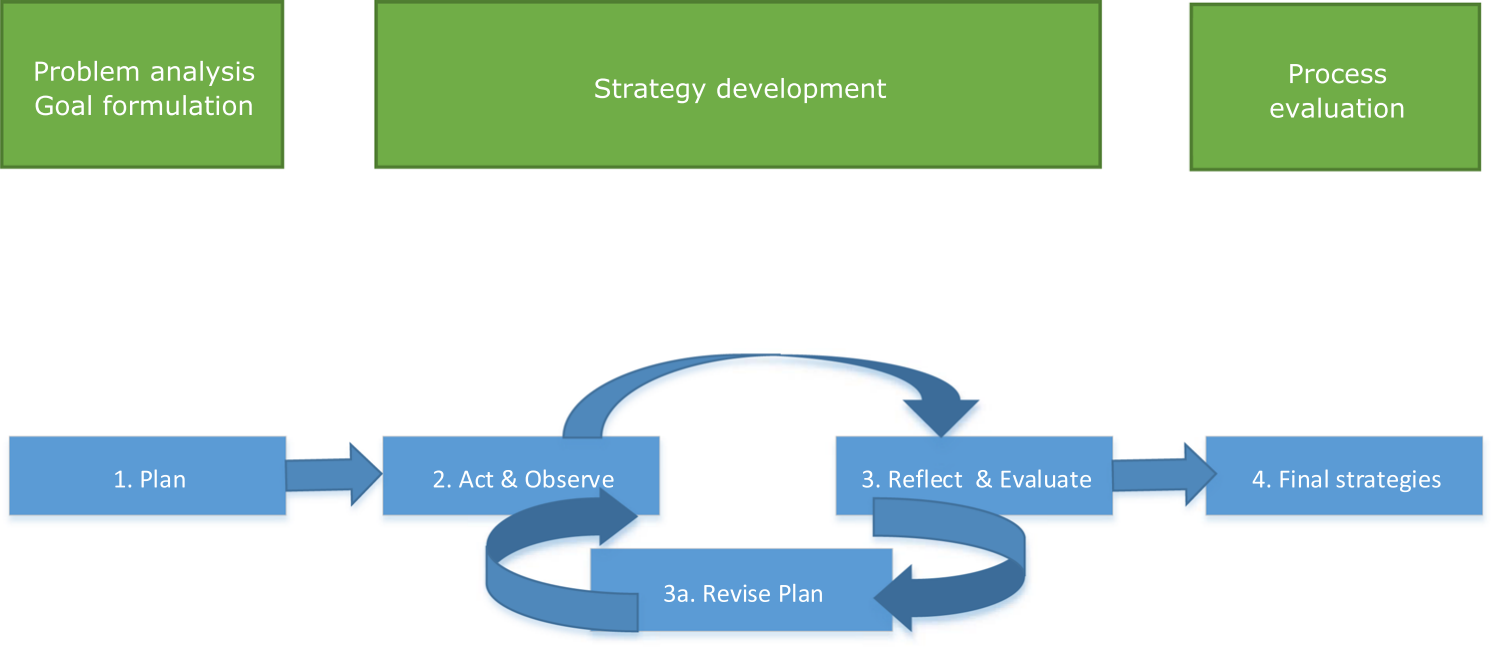
Steps taken during our participatory action research design

This study is part of a larger research project in which we systematically developed and evaluated an implementation strategy process together with all relevant stakeholders by means of a participatory action research design. This bigger study is composed of four smaller studies. The steps that have been taken are depicted in Figure 1.

In Study 1, we identified several implementation barriers at SGL.^19^ On the basis of the identified barriers, four goals for improvement were formulated: (1) goal clarity and added value of the PREM; (2) being prepared for the PREM dialogue; (3) successful execution of the PREM and (4) integrated use of outcomes at the micro, meso and macro levels. The process of drafting strategies based on the problem analysis was described in Study 2 (see Figure 1: act and observe).^20^ In this step, all stakeholders were engaged in developing strategies that are specific and tailored to the facilities' available resources and implementation context.^21^, ^22^, ^23^ These stakeholders included communication‐vulnerable care users, professionals, managers and our research team. Stakeholders then tested strategies, reflected on the application and evaluated the strategies. This provided information to revise the strategies. This cycle was repeated until a consensus among the included stakeholders was reached. Study 3 provided insight into the impact of each stakeholder on the final strategies.^24^

The process evaluation we describe in this article is the fourth and last step of our participatory action research design. We evaluated the uptake, experiences and perceived impact of the tailored implementation strategies. These strategies are aimed at improving the formulated goals, that is, goal clarity, preparation, execution and the integrated use of outcomes of the PREM in a disability care organization. We formulated the following questions:

Question 1a: To what extent are the implementation strategies applied as intended in terms of fidelity, dose, adaptations and reach?
Question 1b: What are the experiences of care users, professionals and facility managers with the tailored implementation strategies, and which factors contribute or hinder implementation uptake?
Question 2: How do stakeholders perceive the impact of the set of implementation strategies on the use of the PREM ‘Dit vind ik ervan!’ as an integrated measure at the micro, meso and macro levels?

## 2 METHOD

### 2.1 Design

We conducted a prospective process evaluation using a mixed‐methods approach based on the Medical Research Council Process Evaluation Framework,^25^ which guides the conduct and report of process evaluations of complex interventions. We used this framework because of the complex nature of both the qualitative PREM and the set of 11 implementation strategies to improve PREM uptake and integrated use of the outcomes. In this framework, the focus of the process evaluation of the implementation is on the delivery of the strategies in terms of fidelity (the extent to which the strategies were provided as intended), dose (exposure), adaptations and reach (number of participants). The study had a concurrent mixed‐methods design in which both components (quantitative and qualitative) are performed simultaneously.^26^

### 2.2 Setting and participants

The study took place at SGL, a Dutch disability care organization offering daily activities, treatment, supported living and living arrangements to people with severe (acquired) intellectual and developmental disabilities, mostly people with acquired brain injuries. SGL has 18 facilities spread out over the Dutch province of Limburg.

A policy officer at SGL purposively selected 3 of the 18 facilities. These facilities were spread out over the province to include multiple context variables, for example, facility management and culture and challenges faced by care users. Facilities had not contributed in earlier phases of the research project, to prevent knowledge bias.^19^, ^20^, ^24^ To safeguard the inclusion of a variety of care users, facilities had at least 12 care users. All care users living at the selected facilities were invited to participate in the study. Professionals and managers had to have worked at SGL for at least 1 year to be familiar with the PREM. Professionals also needed to be case managers of at least one care user, thus responsible for conducting the PREM.

To evaluate strategy uptake at the macro level, we selected representatives of the organization. We involved a manager of the SGL organization, a regional leader, a PREM trainer and a care user representative. All were actively involved in the planning and implementation phase.

### 2.3 Implementation strategies and process

**Figure 2:**
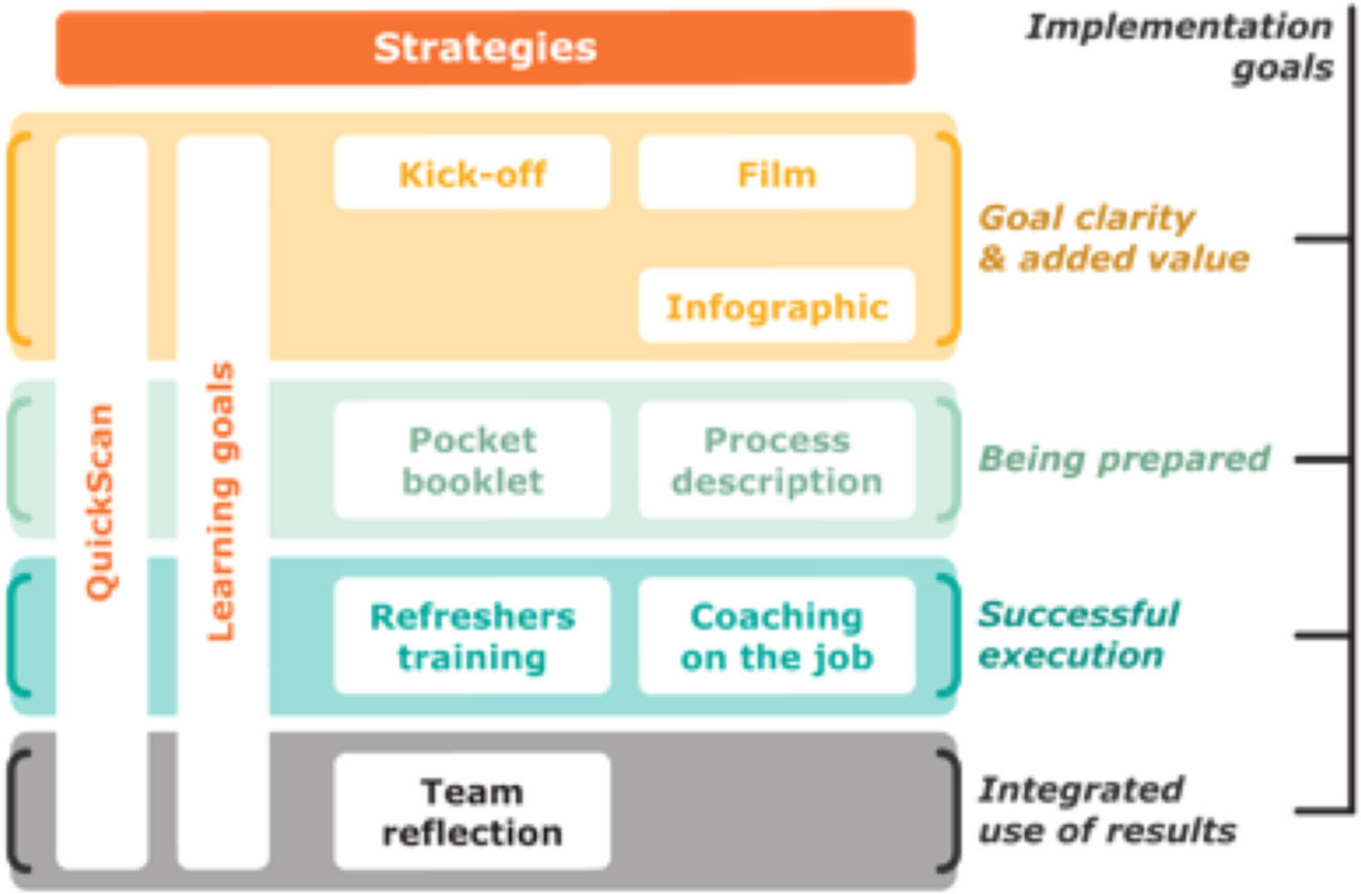
Implementation strategy process

Ten implementation strategies (see Table 1) were executed by SGL to reach the four implementation goals: (1) goal clarity and added value of the PREM; (2) being prepared for the PREM dialogue; (3) successful execution of the PREM and (4) integrated use of outcomes at the micro, meso and macro levels. The implementation strategy process is illustrated in Figure 2.

**Table 1:** Description of the implementation strategies and the users of these strategies

Strategies	Participants	Content	Adaptive
Quickscan	Care users, professionals, facility managers	Questionnaire exploring the state of working with the PREM, tailored to all participants' scope of interest. Quickscans are filled out at the start and the end of the process.	No
Learning goal meeting	Care users, professionals, facility managers	Meeting to formulate facility‐specific learning goals based on quickscan results, using summaries of quickscan results and learning goals guide. Facility‐specific learning goals are added to the second quickscan at the end of the process.	Yes
Kick‐off	Care users, professionals, facility managers, care user representatives	Session to introduce the facility's specific learning goals and implementation strategies, the infographics and film.	Yes
Film	Care users, professionals, facility managers	Short figurative story showing PREMs' added value (https://www.youtube.com/watch?v=hCsRuv3Bz1g&t=8s).	No
Infographic	Professionals, facility managers	Illustration explaining PREMs' goal for care users and explaining PREMs' goal and relation to other used measurements for professionals and facility managers.	No
Pocket booklet	Care users, professionals	A6 booklet to help care users prepare, execute and reflect on PREM dialogue.	No
Process description	Professionals, facility managers	Illustration explaining PREM integration into the annual cycle of care.	No
Refreshers' training	Professionals, facility managers (optional), PREM trainer	2‐h training session addressing facility‐specific learning goals and discussing PREMs' added value, process (using process description) and use (using pocket booklet and addressing communication supportive tools, e.g., talking mats, pictos and pen and paper).	Yes
Coaching on the job	One care user per professional, professionals, PREM trainer	Observation of professionals' PREM execution and provision of feedback to improve PREM execution by the PREM trainer.	Yes
Team reflection	Professionals, complete care team, facility managers	Team reflection on PREM execution and/or outcomes and formulation of potential actions for facilities organized using manual facilitating reflection.	Yes

Abbreviation: PREM, patient‐reported experience measure.

First, the quickscan (Time 0 [T0]) was filled out to guide the formulation of facility‐specific learning goals during a learning goal meeting. A kick‐off meeting was organized to show an introduction film of the PREM and to provide an infographic. The professionals attended a refreshers' training, which was tailored to facility‐specific learning goals. The PREM trainer asked in‐depth questions to clarify the ‘why’ behind a learning goal. Furthermore, the trainer used different techniques, such as role modelling, to improve the skills of the professionals. Moreover, they received a process description to further explain the PREM. Furthermore, the pocket booklet was handed out to professionals. The professionals could introduce this booklet to care users as a way to prepare themselves for the dialogue. During this dialogue between professionals and care users, coaching on the job took place in which the coaching was adapted to the professionals' learning points. Professionals also organized a team meeting to reflect on the execution of the PREM and improvements made to the learning goals. Finally, care users, professionals and team managers filled out the quickscan again after 4 months (Time 1 [T1]).

### 2.4 Data collection

**Figure 3:**
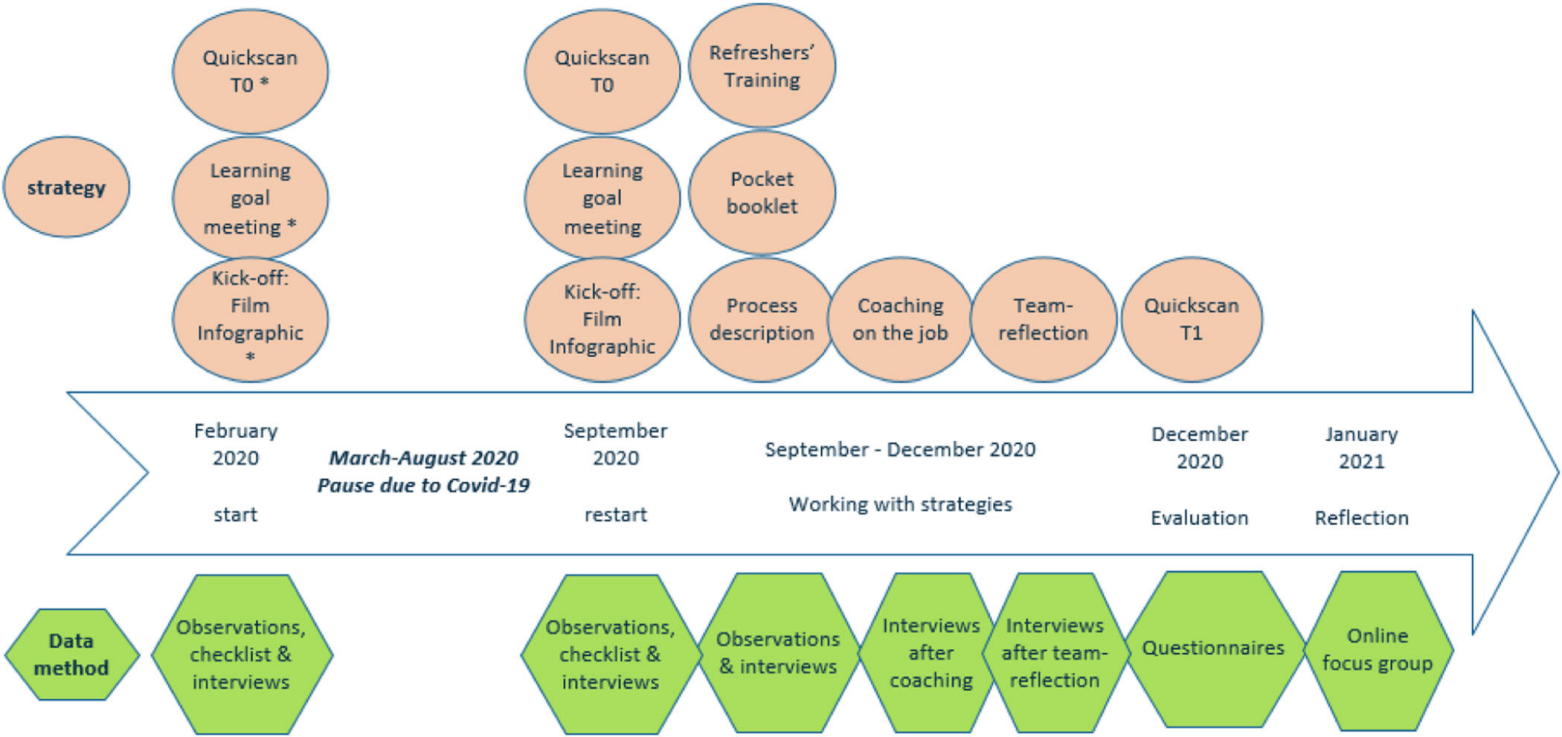
Timeframe for strategies and data collection methods. *These strategies took place in one of the three facilities. In September 2020, the other facilities started. T0, Time 0; T1, Time 1.

Data were collected between February 2020 and February 2021 by means of observations, checklists, semistructured interviews and questionnaires, the quickscans (T0 and T1) and an online focus group interview. See Figure 3 for the timeframe, strategies and data collection methods. In February, one facility started with the implementation. Because of the COVID‐19 pandemic, we had to pause the implementation process between March and September 2020. In September 2020, all participants of the three facilities had completed the quickscan at the start of the implementation (T0). Trained students from nursing, occupational and social sciences assisted care users with filling out the quickscans. They determined the care users' communication vulnerability using the following website: https://www.communicatiekeuzehulp.nl.^27^ (This website was developed by Zuyd University of Applied Sciences, Research Centre of Autonomy and Participation of People with a Chronic Illness. The list is based on the ‘Communication Success Screening’ of Dynavox Mayer‐Johnson; the screening list ‘starten met ondersteunde communicatie?’ by Modem and the developmental model of ‘Taal Centraal’ (2009) of Prof. van Balkom.) It provided information about the current implementation status of the PREM, which was input for the facility‐specific learning goals. These learning goals were added to the quickscan at T1 to evaluate whether goals were reached. With observations and checklists, we observed how the learning goal meeting and kick‐off went. After the kick‐off meeting, learning goal meeting, refreshers' training, coaching on the job and team reflection, we conducted interviews to explore the participants' experiences.

Question 1a: *Strategy applied as intended*. We observed kick‐offs and learning goals meetings to examine participant attendance (reach) and to understand how participants were exposed to the strategy (fidelity, dose, adaptations). Regarding exposure to the refresher training, coaching on the job and team reflections, a researcher (M. v. R.) contacted the trainer or a professional to verify attendance (reach) and to ask how the training went (dose, adaptations).
Question 1b: *Experiences and factors hindering or contributing to strategy uptake*. To explore experiences with strategies and to identify which factors hindered or contributed to the application of the implementation strategies, we conducted semistructured interviews with care users (*n* = 35), professionals (*n* = 11) and facility managers (*n* = 3) at three time points.

First, 1 week after the learning goal meeting and the kick‐off, a researcher (M. v. R.) interviewed care users (*n* = 6) about their experiences. The interviewer used communication‐supportive tools (e.g., icons and pictures). She followed strict COVID‐19 regulations, such as frequent hand‐washing, keeping a 1.5‐m distance and wearing a face mask and gloves. Per the facility, she also interviewed a professional and a facility manager about their experience with the learning goal meeting and the kick‐offs. The professionals and facility managers were interviewed by phone to limit the risks of COVID‐19 spread.

Second, the researcher (M. v. R.) interviewed all professionals and facility managers who took part in the refresher training, coaching on the job and team reflection (*n* = 13) about their experiences, within 2 weeks after the strategies took place. These semistructured interviews by phone started with items that respondents could rate on a scale that ranged from 1 (*poor*) to 5 (*excellent*), such as ‘How would you rate your knowledge about the PREM before the refreshers training?’ This was followed by open‐ended questions to elaborate on each item.

**Figure 4:**
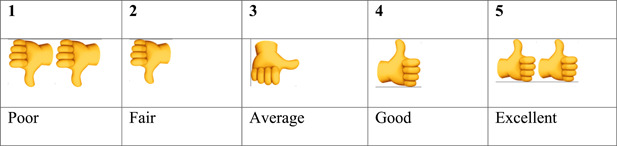
Rating system for care users

Third, after all implementation strategies were applied, all care users (*n* = 35) were interviewed in person about their experiences with the quickscans, the film, the pocket booklet and coaching on the job. The interviews were conducted by trained students from nursing, occupational and social sciences. The interviews started with respondents rating items on a scale that ranged from 1 (*poor*) to 5 (*excellent*), such as ‘How did you experience the kick‐off meeting?’ This was followed by open‐ended questions about each strategy. Care users could use thumbs‐up or thumbs‐down gestures to rank their experiences, as shown in Figure 4.

The interviewers also used reminders—for example, the infographic—to facilitate communication with the care users. All interviews took between 5 and 26 min. COVID‐19 regulations were followed.

Professionals and facility managers (*n* = 14) completed a questionnaire with (scoring/open) questions about their experiences with the quickscan, infographics, film and process description. For example, ‘How would you rate the information load of the infographics?’ ‘How could this be improved?’ In addition, we interviewed the facilitator of the learning goal meetings (*n* = 1) and the trainer who conducted the refresher training and on‐the‐job coaching (*n* = 1). Table 2 provides an overview of the data collection of Questions 1 and 2.

**Table 2:** Data collection evaluation strategy exposure and experiences

Strategy	Qual/quant	Observation	Questionnaire	Interview
Quickscans	Quant	X	X	
Learning goal meeting	Qual/quant	X	X	X
Kick‐off	Quant	X	X	X
Film	Quant	X		
	Qual		X	
Infographic	Quant	X		
	Qual		X	
Pocket booklet	Qual		X	
Process description	Qual/quant		x	X
Refresher training	Qual/quant		X	X
Coaching on the job	Qual/quant		X	X
Team reflection	Qual/quant			X

Abbreviations: Qual, qualitative; Quant, quantitative.

Question 2: *Perceived impact of the set of strategies*. We evaluated the perceived impact of the set of implementation strategies on the four implementation goals in an online focus group. Participants (*n* = 9) were two professionals, three facility managers, a deputy of the board of the SGL organization, a regional leader, a PREM trainer and a care user representative. Beforehand, participants watched a short video presentation as a refresher of all steps of the participatory action research project. A senior researcher (A. J. H. M. B.) moderated the focus group. To facilitate individual input and group discussion, we used an interactive online tool.^28^ For each implementation goal, participants individually determined whether they noticed a negative change, no change or a positive change. This was followed by a discussion of each implementation goal. The focus group took 110 min. It was audio‐recorded and transcribed verbatim.

### 2.5 Data analysis

The quantitative and qualitative analyses were performed by two researchers (M. v. R. and A. v. D.). We analysed independently (1) each data set and (2) each type of participant. Next, we integrated the results of these different analyses as a joint display. This process was guided by the research questions. In the joint display, we integrated results across data sources and different participants. No subanalysis of different participants was carried out.

We used descriptive statistics in Excel 2016 to analyse all scoring questions in the quickscans and interviews and data from checklists. For the analysis of the qualitative data, we used NVivo 12 software.^29^ Open questions in the quickscans and interviews were analysed using directed content analysis, based on whether factors in strategies were experienced as contributing to or limiting PREM application.^30^ First, open‐ended questions and interview transcripts were read to become familiar with the data; then, contributing and hindering factors were coded and categorized. For the analysis of the perceived impact of the strategies, we conducted a deductive analysis by using the four implementation goals as the main categories in the matrix analysis. Relevant text fragments were selected and assigned to the main categories in the analysis framework.

### 2.6 Trustworthiness

To safeguard principles of trustworthiness, we used a variety of methods regarding credibility and transferability. Credibility was enhanced by using prolonged engagement. During the data collection period of 1 year, we became familiar with the setting and context, could test for misinformation and were able to build trust and gain deep insights into the data.^31^ Data triangulation was carried out by using multiple data sources (observations, checklists, questionnaires, interviews and a focus group). In addition, we did a member‐check by presenting study outcomes to participants and asking them for their reflections on the outcomes. Method triangulation took place by using both quantitative and qualitative methods. Investigator triangulation took place given that three researchers were involved in data collection. To enhance transferability, we provided rich data about the setting, sample, data collection and data analysis procedures.^31^

### 2.7 Ethics

All participants in the process evaluation received written, visual and verbal information about the study. We applied communication‐supportive strategies in the information letters, such as the use of short sentences (a maximum of 10 high‐frequency words); one message per sentence and visualizations of the keywords using drawings, photos, icons and bright colours. Participants could ask questions before giving informed consent. Confidential and anonymous handling of data was guaranteed to all participants before data collection. Anonymity was guaranteed through the use of codes. During data collection, participants were given enough time to ask questions. Participants were free to indicate if they wanted to stop or would need a break. The study was reviewed and approved by a research ethics committee.

## 3 RESULTS

### 3.1 Participants

Of all care users of the participating facilities, 47% did not want to participate. Some were hesitant because they found the informed consent process too long or too difficult to understand. Others felt that taking part would be too intense. Nevertheless, they could still join the kick‐off and receive a pocket booklet or infographic. Thirty‐five care users, 11 professionals and 3 facility managers participated in the process evaluation; their characteristics are shown in Table 3. Care users' communication vulnerability is shown in Table 4. Out of the 35 care users, 2 did not experience any difficulties in their communication skills.

**Table 3:** Characteristics of participants engaged in data collection

Characteristic	Care users (*n* = 35)	Professionals (*n* = 11)	Facility managers (*n* = 3)
Participation rate (% of all care users or professionals living or working with ‘Dit vind ik ervan!’ at SGL)	53%	100%	100%
Female, *n* (%)	20 (57)	11 (100)	2 (67)
Age in years, *M* (SD)	51 (13)	39 (15)	54 (6)
Time in years at SGL, *M* (SD)	13 (12)	9 (4)	16 (9)
Education level			
Primary school, *n* (%)	14 (40)	8 (73)	3 (100)
Secondary education, *n* (%)	11 (31)	3 (27)	
Secondary vocational training, *n* (%)	8 (23)		
Higher professional education, *n* (%)	1 (3)		
University education, *n* (%)	2 (6)		

Abbreviation: SGL, Stichting Gehandicaptenzorg Limburg.

**Table 4:** Communication vulnerability level of care users

Communication challenges	Care‐users (*n* = 35)
	*n* (%)
Speaking clearly	16 (46)
Understanding	15 (43)
Remembering	15 (43)
Using pencil and pen	12 (34)
Typing	12 (34)
Speaking loudly	10 (29)
Attention	10 (29)
Seeing	10 (29)
Talking	9 (26)
Signs and facial expression	8 (23)
Hearing	3 (9)
No communication challenges	2 (6)

### 3.2 Findings

Question 1a: *Strategy applied as intended*. The data in Table 5 indicate the extent to which the implementation strategies were applied as intended in terms of reach.

**Table 5:** Reach

Reach	Care users (*n* = 35)	Professionals (*n* = 11)	Facility managers (*n* = 3)
	*n* (%) or %	*n* (%) or %	*n* (%) or %
Quickscan T0	34 (97%)	7 (63%)	3 (100%)
Quickscan T1	31 (89%)	11 (100%)	3 (100%)
Learning goal meeting	100%	100%	100%
Kick‐off with film	28 (76%)	6 (54%)	3 (100%)
Infographics	35 (100%)	11 (100%)	3 (100%)
Pocket booklet	35 (100%)	11 (100%)	3 (100%)
Process description	NA	11 (100%)	3 (100%)
Refresher training	NA	11 (100%)	2 (67%) nonintended
Coaching on the job	NA	11 (100%)	1 (33%) nonintended
Team reflection	NA	9 (82%)	2 (67%)

Abbreviations: NA, not applicable; T0, Time 0; T1, Time 1.

At the start of the implementation (T0), the quickscan was filled out by almost all care users and facility managers and two thirds of the professionals. In the end, three care users did not want to complete the quickscan (T1) because of COVID‐19‐related stress. The outcomes of quickscans (T0) provided input when the facility‐specific learning goals were formulated. These learning goal meetings were attended by at least one care user, one professional and the manager in each facility. Facility managers could invite more care users or professionals. One facility manager invited 14 extra care users. At another facility, two extra professionals attended the learning goal meeting. The kick‐off was tailored to the setting. Two facility managers organized the kick‐off during a regular monthly meeting with care users. In the other facility, there was a special kick‐off night. The majority of care users attended the kick‐offs, watched the film and received an infographic. All professionals participated in the refresher training and on‐the‐job coaching. Even though the refresher training and on‐the‐job coaching were developed for professionals, two facility managers joined as well, and one of them received on‐the‐job coaching. During the refresher training, all attendees received process descriptions and a sufficient number of pocket booklets. They were instructed to hand out the pocket booklets to their care users in preparation for the PREM. One team reflection was cancelled because of COVID‐19 restrictions.

Question 1b: *Experiences and factors hindering or contributing to strategy uptake*. All participants rated their experiences with each implementation strategy on a scale that ranged from 1 (*poor*) to 5 (*excellent*). The results are presented in Table 6. Because of possible memory problems on the part of care users, we asked them what they remembered of the strategy before they shared their experiences. As Table 6 shows, not all care users could remember the film, infographic and pocket booklet, despite their exposure to this strategy. If care users did not remember the strategy, no further questions were asked.

**Table 6:** Results of questionnaires exploring experiences with strategies (between 1 [*poor*] and 5 [*excellent*])

Strategies	Statements	Care users (*n* = 31)	Professionals (*n* = 11)	Facility managers (*n* = 3)
Quickscan	How did you experience the quickscan statements? Mdn (range) How did you experience the quickscan visuals? Mdn (range)	4.5 (4–5)	4 (4–5)	4 (4)
		4 (1–5)	4 (4–5)	4 (4–5)
Learning goal meeting	How did you experience the learning goal meeting? Mdn (range) How did you experience the facilitator during the learning goal meeting? Mdn (range)	5 (5)	4 (4)	4 (4)
		5 (5)	4.5 (4–5)	4 (4–5)
Kick‐off with film	Do you remember the film? *N* (% yes) How did you experience the film? Mdn (range)	21 (59%)	NA	NA
		4 (3–5)	4.5 (4–5)	4 (4)
Infographic	Do you remember the infographic? *N* (% yes) How did you experience the infographic? Mdn (range)	22 (65%)	NA	NA
		4 (1–5)	4(4–5)	4 (4)
Pocket booklet	Do you remember the pocket booklet? *N* (% yes) How did you experience the use of the pocket booklet? Mdn (range)	25 (72%)	NA	NA
		4 (2–5)	5 (4–5)	4 (4–5)
Process description	Do you remember the process description? *N* (% yes) How did you experience the process description? Mdn (range)	NA	NA	NA
			4 (3–5)	4 (4–5)
Refreshers training	How did you experience the refresher training? Mdn (range)	NA	4 (3–5)	4 (4–4.5)
Coaching on the job	How did you experience the coaching on the job? Mdn (range) Would you recommend the coaching on the job? *N* (% yes)	NA	4.5 (4–5)	4 (4)
			11 (100%)	1 (100%)
Team reflection	How did you experience the team reflection? Mdn (range)	NA	4.5 (4–5)	NA

Abbreviations: Mdn, median; NA, not applicable or not asked.

Participants mentioned several contributing factors. First, the active involvement of different stakeholders during the kick‐off and the learning goal meeting was appreciated. Participants valued the combination of input from the quickscan and the contribution of each participant during the learning goal meeting. A care user was surprised to be able to give valuable input during the learning goal meeting: ‘I expected that this would be above my capabilities, but I was able to contribute using my experiences’. A region manager facilitated the learning goal meetings. A facility manager: ‘Especially for care users this must have felt more special because of the region manager facilitating the learning goal meeting. That adds body to the session’.

Second, participants experienced the film, the process description, the infographic and the pocket booklet as practical, providing both an overview of the PREM topics and being an easy way to refresh or transfer knowledge about the PREM within the care team. They mentioned an increased understanding of the goal and value of the PREM due to these tools. The pocket booklet in particular was helpful for care users to prepare the PREM dialogue. A care user: ‘The pocket booklet offered a comprehensive overview of all topics that could be discussed’.

Furthermore, the participants felt that the learning goal meeting, refresher training and on‐the‐job coaching were sufficiently tailored to their context and needs (guided by the location‐specific learning goals). Professionals and facility managers appreciated the focus on practical skills and experienced an increase in their knowledge regarding the why and the how of the PREM. They became aware of their own behaviour and attitude during the PREM dialogue. During on‐the‐job coaching, the coach wrote down sentences as spoken by the professional. Professionals experienced this as confrontational but helpful for reflection. A professional:

I became more aware of how to ask questions without already filling in the care‐user's answer. Before, I quickly started filling in solution‐oriented answers but now I have learnt to let care‐users fill in their own answers. It was nice to have someone say something about that.

Moreover, professionals liked the team reflection because it enabled them to share training and coaching experiences and to transfer knowledge about the PREM dialogue with facility employees who did not join the refresher training and on‐the‐job coaching. A professional: ‘All team members were involved, listened to each other and added personal experiences to the discussion about using the PREM’.

Respondents also experienced several hindering factors for strategy uptake. First, a major hindering factor was implementing the strategies during the COVID‐19 pandemic. We originally planned a 9‐month process evaluation, but we had to pause the implementation process. The evaluation period was reduced to 5 months. Participation became challenging because of restrictions and care users and professionals who tested positive for COVID‐19. This complicated the organization of the refresher training, on‐the‐job coaching and team reflection.

A second experienced barrier was related to the care users' communication challenges. Some care users found it difficult to understand the whole discussion during the learning goal meetings or the kick‐off. Some struggled to express themselves because of the group size. Even though the quickscan, infographic and pocket booklet had been developed together with care users who were communication vulnerable, some care users found it still difficult to read and suggested some improvements. They preferred bigger characters in the infographic and pocket booklet. Furthermore, they asked for an iPad version of the pocket booklet to help care users who could not skim pages because of a physical disability. A care user: ‘It would be helpful to have the pocket booklet on my iPad. Then I would have been able to increase the font size’.

Question 2: Perceived impact of the set of strategies. Guided by the outcomes of the quickscan (T0), all facilities developed three learning goal topics. These facility‐specific learning goals were added to the quickscan at T1 to evaluate whether they had been met. The stakeholders responded as follows.

In the first facility, the learning goals were (1) understanding differences between the measurements used at SGL, (2) knowing and sharing the care‐users' specific needs to perform the PREM and (3) discussing PREM outcomes and experiences with PREM execution in team meetings. These learning goals were met: range = 83%–100%, median = 100%.

In the second facility, the learning goals were (1) knowing care users' individual needs to perform the PREM, (2) care users and professionals do no longer experience the PREM as a pointless task and (3) professionals ask follow‐up questions to better understand the scores of the care users on the PREM. These learning goals were met: range = 87%–100%, median = 91%.

Topics at the third facility were (1) care users know a week in advance that the PREM will take place and have the opportunity to prepare themselves using a pocket booklet, (2) a successfully performed PREM does not necessarily need to be translated in an action items list and (3) PREM reports are discussed with the care users if they want to. In the third facility, the facility‐specific learning goals were met: range = 50%–100%, median = 100%.

In the online focus group, we evaluated the experienced impact of the set of strategies on the four implementation goals. The first goal, ‘Purpose, clarity, and added value’, was unanimous positively evaluated. The attitude of the team toward the PREM had changed, according to the participants. Professional: ‘It is no longer a pointless task’. The representative of the care users shared enthusiastic stories that she had heard from other care users: ‘I noticed a positive change from care users I've spoken with, which made me happy…. I think because now all people know better what “Dit vind ik ervan!” is, we're all facing the same direction’. Facility managers and professionals experienced an increase in sharing the ‘Purpose clarity and added value’ due to the refresher training. The pocket booklet was experienced as very helpful for preparing the PREM dialogue for both care users and professionals. These results had a positive impact on the second implementation goal: ‘Being prepared’ in a way that both care users and professionals felt ready to engage in a dialogue about the care. The perspectives on the impact of the strategies on the third goal, ‘Successful execution’, varied. On the one hand, professionals desired to learn more about conversation techniques to improve PREM dialogue execution. Professional: ‘I have learnt more about using conversation techniques’. On the other hand, professionals still found it challenging to plan and execute the PREM in a limited amount of time.

Professional: It is still difficult to plan the execution of the PREM. During a dialogue, you write down things care users say, but then you have to put it in the electronic care users' files. This file needs to be evaluated. All these steps together are a lot for some care users.

According to the professionals, 5 months of the process evaluation were too short to conduct a PREM with all care users.

In the focus group, the perspectives on the fourth implementation goal, ‘Integrated use of outcomes’, varied. Professionals experienced an improvement in sharing PREM outcomes with care users. They discussed action items with the care users and registered this in the daily care plans. On the team level, professionals perceived an opportunity to discuss outcomes in a team meeting.

Professional: Previously, outdated reports were read like an eight o'clock newsreader, and for the team there was no value in it. I think we should discuss the outcomes among ourselves more frequently in the teams, because this is part of what we do!

SGL management missed feedback from facility managers and could not yet see results at the organizational level.

Overall, focus group participants felt that the strategies created a beginning for a sustainable uptake of the PREM in their organization; however, using the PREM on a macro level has been considered a ‘work in progress’. Their expectant attitude was expressed by one of the managers: ‘If facilities with positive PREM experiences share those experiences in different ways with other facilities, they will be stimulated. This way the speed of sharing positive experiences can be increased’.

## 4 DISCUSSION

This study aimed to evaluate the extent to which the strategies were applied as intended, the experiences regarding tailored implementation strategies and the perceived impact of this set of strategies on the integrated use of a qualitative PREM in disability care. The process evaluation took place at three facilities of a Dutch disability care organization. The reach of the strategies to the care users, professionals and managers was acceptable—between 76% and 100%. The participants valued the tailored approach, which enabled them to focus on facility‐specific learning goals. Hindering factors were complications in the planning of the strategy rollout because of COVID‐19 and the communication vulnerability of care users. The perceived impact of the set of implementation strategies was noted mainly at the micro level, improving goal clarity and added value and preparation of the PREM.

This process evaluation was the last step of a participatory action research project in which we developed implementation strategies in continuous cocreation with all relevant stakeholders, including communication‐vulnerable care users.^19^, ^20^, ^32^ This research approach enabled us to generate both research knowledge and knowledge for practice, which could immediately change practice in a positive way. Stakeholders were involved at the micro level (care users, professionals and PREM trainers), the meso level (professionals, PREM trainers and managers) and the macro level (managers and quality advisors). This stakeholder engagement and the use of continuous iterations may have strengthened the uptake and experiences with the implementation. The participants found that the strategies aligned with their practice and provided an answer to problems they face. In particular, the combination of fixed and adaptive strategies was appreciated because the adaptive strategies could be tailored to the facility‐specific learning goals. This seems to be a promising and feasible approach that promotes implementation. This approach can be replicated by other organizations, but only after context‐specific problem analyses and the selection of strategies. Other organizations working with the ‘Dit vind ik ervan!’ PREM can use the quickscan to determine their learning goals and improve implementation in their organization with the available strategies.

Because the region manager was involved as a facilitator during the learning goal meetings, one felt the support and importance of improving the PREM within SGL. This highlights that implementation uptake highly depends on our systematic approach and involvement of stakeholders from all levels in the organization, and needs to be embedded and embraced.^22^, ^23^, ^33^

Even though our implementation strategies were cocreated,^19^, ^20^, ^32^ some care users with communication challenges still expressed difficulties working with some of the strategies. They gave concrete suggestions for improvement, for example, bigger characters in the pocket booklet or an iPad version. This shows that cocreation and tailoring implementation strategies are a continuous process. Koshy et al.^34^ assumed that interventions are never finished and can be adapted over time in accordance with changes in practice or context. Thus, cocreation is significant for making plans and developing strategies, and valuable suggestions for the adaption of the implementation strategies can be obtained after evaluation.^14^, ^35^ In this regard, Bentzen^35^ showed that ownership of strategies and strategy outcomes are strengthened if cocreation continues in later stages of implementation. This underscores the importance of implementers remaining open to feedback provided by the strategies' users.

In our study, the tailoring of strategies (refresher training, on‐the‐job coaching and team reflection) was guided by facility‐specific learning goals. These learning goals were based on outcomes that were derived from the quickscan. Baker et al.^37^ and Lewis et al.^36^ have shown the power of tailoring strategies to contextual factors to improve intervention uptake.

The stakeholders questioned the impact of the tailored implementation strategies on the integrated use of the PREM outcomes. Although professionals experienced that PREM outcomes were more often discussed, and actions were taken with both the care users (micro) and the care teams (meso), the outcomes could not be translated into actions for quality improvements at the organizational (macro) level. This is not surprising because the challenges that were identified during the problem analysis mainly addressed the micro level.^19^ In this regard, Foster et al.^14^ recommended starting with planning the organizational aspects to administer the PREM and preparing the staff for PREM use. Professionals need to be convinced about the value of the PREM at the micro level. The next step is to use the results of the PREM at the meso and macro levels.

### 4.1 Strengths and limitations

A strength of this study is the use of diverse qualitative methods (observations, checklists, questionnaires, interviews and focus groups) and quantitative data to gain insight into the adoption and experience of the strategies.

A second strong point is the systematic and stakeholder approach. During the problem analysis and strategy development phase, the research team was actively engaged in the process together with the other stakeholders at SGL. The role of the research team changed from partners to one that involved collecting data and evaluating the process as academic researchers. SGL had full ownership of the implementation. Moreover, the research team had to stay at a physical distance because of COVID‐19. We were still able to collect the information we aimed for by means of phone interviews and web‐based questionnaires. Fortunately, care users could still be supported by filling out questionnaires and the quickscans. This was delegated to independent, trained healthcare students. We only missed nonverbal clues in the collection of data from professionals and managers.

The set of implementation strategies was rolled out in three SGL facilities that were not engaged in the development of the strategies. Nevertheless, because of the 4‐year‐long engagement of the SGL organizations, management perceived a coownership of the strategies. This resulted in no dropouts and the continuous engagement of these three facilities even though the context of COVID‐19 was challenging. This may be a result of all the time and energy that had been invested by the researchers to understand the organization and to involve all the stakeholders in the development process.

This study is also subject to weaknesses. This process evaluation was originally planned for a period of 9 months. Because of COVID‐19, we had to shorten the study to a period of 5 months. Only 53% of all care users living at the three facilities participated in the data collection part of the process evaluation. One reason for this low participation rate is the informed‐consent procedure. To participate in the process evaluation, participants needed to be informed about all the pros and cons of participation. Many care users have communication vulnerabilities, which hindered them in reading through all the legally required 20 pages of informed consent, although this was written and visualized using communication‐supportive methods.^38^ For some care users, this comprehensive document was a barrier to participation. The other complication of including care users was the COVID‐19 situation. Because of the mental impact (e.g., anxiety, stress) of the pandemic, some care users felt discouraged and did not want to take part in a research study.

The findings of our process evaluation are promising regarding better PREM uptake at the micro level. Given the idea that implementation processes depend on a continuous learning process that changes both interventions and organizations,^35^ it would have been valuable to determine the impact on integrated use of PREM outcomes and sustainability of strategies for a longer period of time.

## 5 CONCLUSION

This study of the implementation of a PREM, in which the strategies were cocreated and tailored to the three disability care facilities, shows good uptakes in daily practice. The impact was mainly experienced on the level of the care users and professionals, regarding goal clarity and the added value of the PREM and preparation for the PREM dialogue. More time and effort are needed for the integrated use of the PREM at the meso level to monitor the quality of care and enhance team reflection and at the macro level to facilitate organization‐wide improvements and external reporting about quality of care. The stakeholder engagement in the whole process, from problem analysis to cocreated implementation strategies, may have strengthened the adoption of and experiences with the implementation.

## AUTHOR CONTRIBUTIONS

*Study conception and design*: Marjolein van Rooijen, Anneke van Dijk‐de Vries, Stephanie Lenzen, Albine Moser, Ruth Dalemans and Anna J. H. M. Beurskens. *Data collection*: Marjolein van Rooijen, Anneke van Dijk‐de Vries and Anna J. H. M. Beurskens. *Analysis and interpretation of results*: Marjolein van Rooijen, Anneke van Dijk‐de Vries, Albine Moser and Anna J. H. M. Beurskens. *Draft manuscript preparation*: Marjolein van Rooijen, Anneke van Dijk‐de Vries, Albine Moser and Anna J. H. M. Beurskens. All authors reviewed the results and approved the final version of the manuscript.

## CONFLICT OF INTEREST

The authors declare no conflict of interest.

## ETHICS STATEMENT

The study was reviewed by a Research Ethics Committee of Zuyderland and Zuyd University (METCZ20200001).

## Supporting information

Supporting information.

## Data Availability

The data that support the findings of this study are available from the corresponding author upon reasonable request.
