# Supplementary material for: Implementation of a patient‐reported experience measure in a Dutch disability care organization: A process evaluation of cocreated tailored strategies
Source: Health Expect. 2022 Nov 8;26(1):132–45. doi: 10.1111/hex.13628 (PMC9854305; doi:10.1111/hex.13628)
Supplement: Supplementary file 1 — Supporting information. [file HEX-26--s001.docx]

**Appendix 1**

**This is how I feel about it!**

Appropriate service starts with an open conversation between care-user and professional. What do care-users find important regarding their quality of life? What do they experience as pleasant? Where should there be improvements in care and service? ‘This is how I feel about it!’ is an approach that collects care-users’ experiences. It is developed by and for professionals who are motivated to evaluate the quality of care together with the care-user in order to improve it.

**A dialogue**

The idea behind ‘This is how I feel about it!’ is a conversation between a care-user and/or someone who knows him or her well. Often this the mentor. There are 10 main themes, such as ‘Body’, ‘Participation’ and ‘Family’, which will be discussed by means of an exploratory dialogue. Since the focus is on an open dialogue, there are no set of questions or mandatory checklists per theme. The mentor listens, asks questions, and holds back on his or her own opinion. The care-user gives a grade per theme, explains whether change on this topic is desired, and is asked to prioritize the themes. The gathered information provides input for the yearly evaluation of the individual care plan.


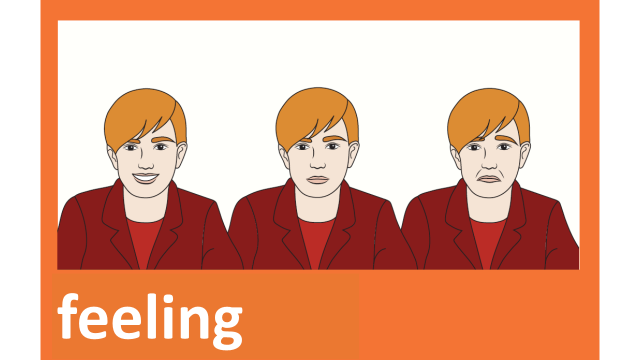

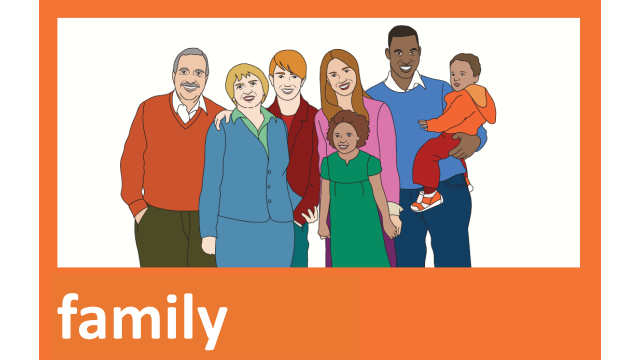


The way in which the conversation takes place depends on the preference and the possibilities of the care-user. There are different variations of ‘This is how I feel about it!’:

- *I speak*: For care-users who are able to express themselves verbally.
- *I see and I speak:*  Using the experience of relatives as a valuable (additional) source of information.
- *I show*. For care-users who are not able to express themselves verbally. In this variation, films are used to understand the non-verbal reactions of the care user.

**Learning together**

Mentors need the right skills and tools for the approach ‘This is how I feel about it!’ They receive training to become professional in facilitating an exploratory dialogue.

The method takes place in the setting where quality really counts: in the relationship between the care-user and care professional. Professionals can apply changes on an individual level. Moreover, the (anonymous) information derived from the conversations with care-users can be used within the organization as a motivator for reflection, development and innovation. Additionally, the information is suitable for external reports about the quality of care.
